# The Effect of Ethyl Alcohol upon Pedestrian Trauma Sustained in Traffic Crashes

**DOI:** 10.3390/ijerph16081471

**Published:** 2019-04-25

**Authors:** Witold Pawłowski, Dorota Lasota, Mariusz Goniewicz, Patryk Rzońca, Krzysztof Goniewicz, Paweł Krajewski

**Affiliations:** 1 Department of Disaster Medicine, Medical University of Warsaw, 02-091 Warszawa, Poland; witold.pawlowskl@dr.com; 2 Department of Experimental and Clinical Pharmacology, Medical University of Warsaw, 02-091 Warszawa, Poland; 3 Department of Emergency Medicine, Medical University of Lublin, 20-059 Lublin, Poland; mariusz.goniewicz@gmail.com (M.G.); patryk.rzonca@umlub.pl (P.R.); 4 Department of Security Studies, Polish Air Force Academy, 08-521 Dęblin, Poland; k.goniewicz@law.mil.pl; 5 Department of Forensic Medicine, Medical University of Warsaw, 02-091 Warszawa, Poland; pawel.krajewski@wum.edu.pl

**Keywords:** ethyl alcohol, traffic crash, pedestrians

## Abstract

Introduction: Every year more than 1.2 million people worldwide die due to trauma sustained in road crashes, with an additional number of people injured exceeding 50 million. To a large extent, this applies to so called “unprotected road users”, including pedestrians. The risk involved in a traffic crash for pedestrians can result from many factors, one of which is participation in road traffic when under the influence of alcohol. The aim of this study was to analyze the impact of alcohol use among pedestrians as unprotected road traffic participants, and the consequences of them being struck by motor vehicles. Material and methods: The source of data was the medical documentation of the Department of Forensic Medicine at the Medical University of Warsaw. The sample for this research consisted of 313 pedestrians who were victims of fatal road crashes resulting from a collision with a mechanical vehicle. The obtained results were subjected to statistical analysis using the STATISTICA version 12.5 program (StatSoft Polska, Cracow, Poland). Results: Male fatalities constituted the majority of the study sample. Nearly half of the fatal pedestrian victims were found to be under the influence of alcohol. The statistical analysis demonstrated a significant relationship between the gender and age of the victims, as well as between the place of the event, the place of death, the mechanism of the event, and the presence of alcohol in pedestrians. Conclusions: Among pedestrians, victims of road crashes who were under the influence of alcohol were predominantly drunk young males. Victims under the influence of alcohol were more likely to become fatalities in crashes where the mechanism of the incident was being struck by a passenger car, and when the place of the incident was a rural area, in these cases the rates of death directly at the scene were much more frequent. The eradication of alcohol consumption by all road users should be the overriding objective of all measures aimed at reducing the number of road crashes.

## 1. Introduction

Road transport is one of the most complex and dangerous systems encountered by people on a daily basis. Around 1.2 million people worldwide die as a result of road crashes every year, and more than 50 million people are injured. According to estimates provided by the World Health Organization (WHO), without the implementation of new initiatives to improve road safety by 2020, the number of deaths and injuries resulting from road crashes will increase by 65%, while in low- to middle-income countries this increase could reach up to 80%. To a large extent, this applies to “unprotected road users” (i.e., pedestrians, cyclists, motorists, and motorcyclists), who account for 46% of the global percentage of fatalities resulting from road crashes (in Europe 38%, in Poland 39%) [1,2,3,4,5,6].

A disproportionately large number of crashes involving vulnerable road users occur in low-income countries. Nonetheless, the consequences of this also create an enormous burden for the societies of industrialized countries. The risk of a traffic crash for vulnerable road users can be influenced by many factors, the most important of which is the design and construction of communication systems from the perspective of motor vehicle users. Other risk factors for this group of road users include, among others, poor visibility of pedestrians and cyclists, non-compliance with road safety rules, inappropriate design of the front of the car, and participation in road traffic after alcohol consumption [1,7,8,9].

The influence of alcohol on the drivers of motor vehicles has been studied for decades. Numerous studies have indicated that alcohol consumption is a significant risk factor impacting the incidence of road crashes, which also significantly increases the risk of death in relation to road traffic participants in whom alcohol is not found [1,7,8,9]. The influence of alcohol on unprotected road users has not been so widely analyzed, and there are few publications which deal with this issue [9,10]. The aim of this study is to analyze the impact of alcohol use among pedestrians as unprotected road traffic participants, and the consequences of them being struck by motor vehicles.

## 2. Materials and Methods

The source of data was the medical documentation of the Department of Forensic Medicine (polish ZMS) of the Medical University of Warsaw. After verification of the aforementioned documents, 313 pedestrians qualified for the study as fatalities of a motor vehicle collision, out of all of the victims of road crashes that were recorded in the Department documentation in the examined period. The analysis included parameters such as the gender and age of victims, the types of injuries, the mechanism of the crashes, the place of the event, and the place of death recorded for the analyzed individuals.

The acquired data were collected in a Microsoft Excel database of an MS Office 2016 package for Windows 7. The obtained results were subjected to statistical analysis using the STATISTICA version 12.5 program (StatSoft Polska, Cracow, Poland). The quantitative data were presented as mean and standard deviation, whereas the qualitative data were presented as numbers and percentages. A Chi2 test was used to assess significant differences between the qualitative variables. To examine the differences between two groups, the Mann–Whitney U-nonparametric test was used. The level of significance was set at *p* < 0.05.

The research project was approved by, and received a positive opinion from, the Bioethical Commission of the Warsaw Medical University (No. AKBE/112/14).

## 3. Results

Of the study population, 208 were men (66.45%). The age of the victims varied, ranging from 3 to 91 years, with the average age being 68.5 (±12.70) years. Most often, victims who were struck by a motor vehicle included people aged between 45 and 64 (36.10%). Alcohol was found in 162 of the victims (51.76%). The majority (276) of pedestrians who were struck experienced multiple injuries (88.18%), death occurred at the crash site for 211 of the victims (67.41%), and 212 victims were from crashes where the mechanism involved was a car (67.73%). Detailed results are presented in Table 1.

The conducted statistical analysis highlighted a significant relationship between gender and the age of the victim, the place of the event, the place of death, and the mechanism of the event, in relation to the presence of alcohol in the blood of the victims. The presence of alcohol was more often found in men (83.33%), among victims aged 45–64 years (49.38%), and in cases where the place of the incident was a rural area (51.23%). Victims under the influence of alcohol were more likely to die in crashes in which the mechanism of the incident was being struck by a car (74.69%), and in these cases death occurred directly at the scene more often (82.10%). 

There was no statistically significant relationship between the presence of alcohol and the type of injury sustained. Detailed results are presented in Table 2.

**Figure 1:**
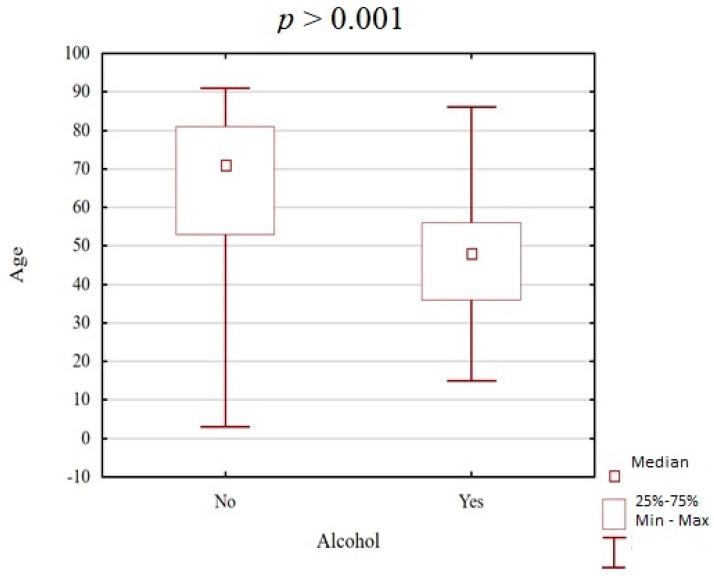
Analysis of the relationship between the age of the victims and the presence of ethyl alcohol in their systems.

The statistical analysis revealed that victims who were under the influence of alcohol were significantly younger (45.81 years) than those who did not test positive for alcohol (62.96 years) (Figure 1).

A statistical analysis taking into account the concentration of alcohol in the victims that were struck by a motor vehicle demonstrated that a higher concentration occurred among men, when the place of the event was a rural area, and in a situation where the death occurred directly at the scene (*p* < 0.05) (Table 3).

## 4. Discussion

Injuries resulting from traffic crashes, despite the actions undertaken with regard to the improvement of road safety, still constitute a serious health and economic problem in Poland, as well as in other European Union countries. Injuries resulting from high-energy mechanisms impacting upon the human body during a road crash might be a direct cause of death or long-term specialist treatment. Unprotected road users are particularly susceptible to heavy, multiple, and multi-organ injuries [3,5,10].

The results of this research demonstrated that victims who had been hit by a car in the analyzed material were mainly men aged 45–64 years, and the highest rates of these types of incidents were recorded in rural areas. Research by de Carvalho Ponce et al. on the impact of alcohol in traffic crashes in Sao Paulo showed that the victims of road crashes were mostly men aged 25–34 years [11]. Similar results to de Carvalho Ponce et al. were noted by Gjerde et al. (2011), who analyzed the impact of alcohol and psychoactive substances on road crashes in Norway [12], as well as Hickox et al., who analyzed pedestrian mortality in Clark County, Nevada [13]. De Boni et al., in their research on factors related to alcohol consumption and drugs by participants of road crashes in southern Brazil, demonstrated that victims being hit by cars were also mainly men, with the average age being 37 years [14].

In studies concerning gender, age, and alcohol concentration among victims hit by a car, Holubowycz demonstrated that among all road users, it was pedestrians who were characterized as having the highest concentration of alcohol levels in their blood [15]. The analysis presented by de Carvalho Ponce et al. indicated that the occurrence of alcohol was present more frequently in men aged between 25 and 44 years. However, in the group of pedestrians who were road crash victims, alcohol was found in over one third of the victims who were hit by a car [11]. De Boni et al. revealed in their studies that men, middle-aged people, and car drivers were more likely to be under the influence of alcohol [14].

The results of our research demonstrated that those under the influence of alcohol were more often men aged 45–64 years. The most common place for such incidents was rural areas, and victims who were under the influence of alcohol were more likely to die in situations which involved being struck by a passenger car, with death frequently occurring at the scene of the event.

Alcohol is the most widely consumed legal intoxicant and psychoactive substance in the world. Even a small concentration of alcohol impacts the perception and behavior of those who consume it. The state after alcohol consumption leads to a greater risk of road crashes and suicides worldwide [11,12,14,16,17,18,19,20].

The results of our research demonstrated that the average alcohol level of victims was 2.05 mL/mL. It was also evident that those under the influence of alcohol were much younger than other fatalities. In addition, it was found that a higher concentration of alcohol occurred in male victims, where the place of the incident was a rural area, and in a situation where the death occurred directly at the scene of the event. Research from de Carvalho Ponce et al. confirmed that the pedestrian group was the only one which revealed a statistically significant difference in the mean age and the presence of alcohol. These studies show that victims who were found to be under the influence were significantly younger than those with negative results [11]. A study by Dultz et al. researching the impact of alcohol consumption among pedestrians struck in motor crashes in New York showed that 14.3% of all pedestrians tested positive for alcohol and that men were more likely to be under the influence of alcohol than women [21]. Rao et al., in their studies on drivers and pedestrians who died as a result of road crashes, demonstrated that the average alcohol level was 2.34 ml/mL in pedestrians, which was significantly higher than that of drivers. A positive result concerning the presence of alcohol was significantly more frequent in men, and the average age was 43 years [22]. Similar results were presented by Lasota et al. in their research on the impact of intoxication on the risk of traffic crashes. Here, the average alcohol concentration in fatal pedestrian traffic crash victims (2.04‰) was significantly higher than the average alcohol concentration in car users (1.46‰) [23,24].

## 5. Limitations

The presented results have some limitations. This research only included fatalities from being hit by a car, and those which had been subjected to autopsy. Due to the essence of the problem, which is excessive alcohol consumption and its related consequences, it seems necessary to conduct further, in-depth research in this area.

## 6. Conclusions

Ethyl alcohol is an important risk factor in road crashes. Injuries resulting from high-energy mechanisms which impact pedestrian traffic participants are usually the direct cause of death or long-term specialist treatment.

This research demonstrates that among pedestrians, victims of road crashes in which alcohol was present were predominately drunk young men. Victims under the influence of alcohol were more likely to die in crashes where the mechanism of the incident was being hit by a passenger car, and when the place of the incident was a rural area. Under these circumstances, death at the scene of the crash was also a far more frequent occurrence.

Any endeavor in the form of social campaigns on road safety which aims to prevent traffic crashes should have one common goal—the elimination of alcohol by all road users.

## Figures and Tables

**Table 1:** Characteristics of the studied population.

Characteristics	
**Gender *n* (%)**	
Female	105 (33.55)
Male	208 (66.45)
**Place of the event *n* (%)**	
Urban area	191 (61.02)
Rural area	122 (38.98)
**Age (years) *n* (%)**	
0–17	4 (1.28)
18–44	98 (31.31)
45–64	113 (36.10)
65+	98 (31.31)
Mean (SD)	68.5 (12.70)
**The presence of ethyl alcohol *n* (%)**	
Yes	162 (51.76)
No	151 (48.24)
Average concentration of ethyl alcohol (SD)	2.05 (0.90) * 0.98 (0.43) **
**Type of traumatic injuries *n* (%)**	
Multiple trauma	276 (88.18)
Isolated/other	37 (11.82)
**The place of death *n* (%)**	
Death at the scene of the event	211 (67.41)
Death in the first day of hospitalization	102 (32.59)
**Mechanism of the crash *n* (%)**	
Car	212 (67.73)
Cargo/delivery/bus	53 (16.93)
Rail vehicle	48 (15.34)

* Ethyl alcohol concentration in blood, muscles, and vitreous bodies expressed in promiles (‰); ** value of ethyl alcohol concentration expressed in milligrams per cubic decimeter (mg/dm^3^).

**Table 2:** Analysis of the relationship between the presence of alcohol in fatalities and gender, age, place of event, mechanism of the event, type of injury, and the place of death.

Characteristics	The Presence of Ethyl Alcohol		
	Yes *n* (%)	No *n* (%)		
**Gender**			*p* < 0.001	
Female	27 (16.67)	78 (51.66)		
Male	135 (83.33)	73 (48.34)		
**Place of the event**			*p* < 0.001
Urban area	79 (48.77)	112 (74.17)	
Rural area	83 (51.23)	39 (25.83)	
**Age (years)**			*p* < 0.001
<18	1 (0.62)	3 (1.99)	
18–44	68 (41.98)	30 (19.87)	
45–64	80 (49.38)	33 (21.85)	
>65	13 (8.02)	85 (56.29)	
**Mechanism of the crash**			*p* < 0.05
Struck by a car	121 (74.69)	91 (60.26)	
Struck by cargo/delivery/bus	21 (12.96)	32 (21.19)	
Struck by rail vehicle	20 (12.35)	28 (18.54)	
**Type of traumatic injuries**			*p* < 0.05
Multiple trauma	143 (88.27)	133 (88.08)	
Isolated/other	37 (11.73)	18 (11.92)	
**The place of death**			*p* < 0.001
Death at the scene of the event	133 (82.10)	78 (51.66)	
Death in the first day of hospitalization	29 (17.90)	73 (48.34)	

**Table 3:** Analysis of the relationship between alcohol levels and gender, place of event, and place of death.

Variables	Concentration of Ethyl Alcohol * Mean (SD)	*p*
**Gender**		
Female	1.69 (0.95) 0.81 (0.45) **	*p* < 0.05
Male	2.12 (0.88) 1.00 (0.42) **	
**Place of the event**		
Urban area	1.86 (0.95) 0.88 (0.45) **	*p* < 0.05
Rural area	2.23 (0.82) 1.07 (0.39) **	
**The place of death**		
Death at the scene of the event	2.11 (0.91) 1.00 (0.43) **	*p* < 0.05
Death in the first day of hospitalization	1.77 (0.84) 0.83 (0.41) **	

* Ethyl alcohol concentration in blood, muscles, and vitreous bodies expressed in promiles (‰); ** value of ethyl alcohol concentration expressed in milligrams per cubic decimeter (mg/dm^3^).
